# Operational Consideration and Best Practices for Implementation of an Early Alzheimer's Disease Patient Care Pathway

**DOI:** 10.1002/alz70861_108093

**Published:** 2025-12-23

**Authors:** Babak Tousi, Feride H Frech, David S. Geldmacher, Jeff Vawter, Pierre N. Tariot, Cate Polacek, Yuval Zabar, Christine Divers, Hideki Toyosaki

**Affiliations:** ^1^ Lou Ruvo Center for Brain Health, Cleveland Clinic, Cleveland, OH USA; ^2^ Eisai Inc., Nutley, NJ USA; ^3^ University of Alabama at Birmingham, Birmingham, AL USA; ^4^ Premier, Inc, Charlotte, NC USA; ^5^ Banner Alzheimer's Institute, Phoenix, AZ USA; ^6^ Biogen Inc, Cambridge, MA USA

## Abstract

**Background:**

Timely Alzheimer’s disease (AD) diagnosis and treatment are public health priorities. Barriers to accessing innovative diagnostics and therapies prevent Americans with AD from benefiting from disease‐altering interventions. Understanding barriers and facilitators of implementing an early AD patient care pathway may improve patient care and outcomes.

**Methods:**

An evidence scan and expert council, a panel of healthcare providers, were employed to understand current AD pathways, incorporating empirical evidence and real‐world expertise. The evidence scan searched MEDLINE, CINAHL and websites from relevant organizations and health systems. The evidence council, a panel of healthcare professionals from diverse AD‐related specialties with broad geographic representation, shared their pathway development experiences and best practices. These data were summarized into operational considerations and best practices to facilitate the development and implementation of an Early AD Patient Care Pathway.

**Results:**

The evidence scan identified biomarker and digital algorithms used for risk stratification, early detection, timely diagnosis, and preventive and therapeutic interventions as operational considerations for AD pathway development. The expert panel recognized best practices including (1) conducting first‐line identification in primary care, second‐line with specialists; (2) incorporating shared decision‐making; (3) utilizing nurse (or other) navigators; (4) updating medical record systems; and, (5) leveraging advanced practice providers to improve patient tracking and care coordination and identifying patients’ resource needs and community resources. The evidence council highlighted additional considerations including the need for more patient‐friendly education (e.g., videos, infographics, culturally sensitive education) and language interpretation/translation, and improved collaboration with imaging and infusion partners.

**Conclusions:**

AD care pathways may facilitate timely diagnosis and early intervention for patients with AD, potentially leading to improved care coordination and patient safety. Early feedback suggests health systems anticipate increased efficiency in healthcare resource utilization/capacity and a positive impact on patient satisfaction. Future projects should evaluate AD pathway implementation and their impact on patients and health care organizations.

